# The Effect of Radiative Cooling on Reducing the Temperature of Greenhouses

**DOI:** 10.3390/ma11071166

**Published:** 2018-07-09

**Authors:** Chia-Hsin Liu, Chyung Ay, Jo-Chuan Kan, Maw-Tien Lee

**Affiliations:** 1 Department of Applied Chemistry, National Chia Yi University, Chiayi City 60004, Taiwan; s1060341@mail.ncyu.edu.tw (C.-H.L.); s1050364@mail.ncyu.edu.tw (J.-C.K.); 2 Department of Biomechatronic Engineering, National Chia Yi University, Chiayi City 60004, Taiwan; cay@mail.ncyu.edu.tw

**Keywords:** covering material, passive cooling, near infrared radiation

## Abstract

Currently, greenhouses are widely used for the cultivation of various crops. However, in tropical and subtropical regions, undesired near-infrared radiation (NIR) causes heat loads inside the greenhouse. Recent works have demonstrated that radiative cooling, releasing energy via radiative heat exchange where the heat is dumped directly into outer space, can be achieved by using silica particles designed to emit in the infrared atmospheric transparency window. The purpose of this study is to improve the plastic greenhouse cladding to regulate the temperature inside the greenhouse, mainly by passive cooling. Low-density-polyethylene (LDPE)-based formulations with anti-fogging agent, UV stabilizer, and silica particles were prepared by the melt blending technique and were formed into a double film by extrusion molding. Experimental results showed that under 35 °C ambient conditions, the inner temperature of the simulated greenhouse with the newly developed cladding was 4 to 5 °C less than that of the greenhouse with the commercial agricultural polyethylene (PE) film.

## 1. Introduction

A greenhouse is an artificial architecture which protects crops from adverse weather conditions, such as rain, wind, heat burn, insects, and diseases, to increase the yields [1,2,3]. Solar radiation, consisting of 5% ultraviolet radiation (UV, 100–400 nm), 46% visible radiation (VIS, 400–780 nm), and 49% near-infrared radiation (NIR, 750–2500 nm) transmits into a greenhouse through the cladding to supply the crops for photosynthesis. However, only photosynthetically active radiation (PAR, 400–700 nm) is crucial for plant growth and photosynthesis [4]. UV radiation affects film degradation and pollinator behavior, and NIR radiation is a direct source of heat. Much of NIR radiation penetrates into greenhouses and causes heat loads. Low-latitude countries are generally torrid in summer, and overly high temperature inside a greenhouse is harmful to people who work inside and crops that grow.

There were many methods described in literature to regulate the temperature inside the greenhouse, such as ventilation, an evaporative cooling system, and a liquid radiation filter (LRF), etc. [1,4,5,6,7,8,9,10,11,12,13,14,15,16,17,18,19,20,21,22,23,24]. However, these methods may consume plenty of energy. Ventilation can reduce the temperature by exchanging the air between the greenhouse and the ambient environment. However, in extremely hot summer conditions, very hot ambient air cannot provide an adequate cooling effect [1,6]. LFR is used as a selective solar radiation absorber for controlling the greenhouse temperature in a hot climate. LRF strongly absorbs the UV and NIR wave bands and highly transmits PAR. However, it often needs a poisonous CuSO_4_ solution. Moreover, the construction and the operation of LRFs are very expensive [10]. An evaporative cooling system can take away the heat through water evaporation, and the most essential requirement for this system to operate efficiently is the availability of pure and fresh water. Water sources with saline and brackish water will cause the clogging of pads, as a result there is fast deterioration in the cooling performance and the restriction of air flow will lead to increased electrical energy consumption [6]. There are some limits, such as region, cost, and energy consumption for the above methods.

The use of plastics in agricultural applications stretched back to the middle of the 20th century. Improvements in plasticulture technology systems have led to an increase in yields, earlier harvest, better crop protection, and water resource preservation. For the purposes of saving energy, it is feasible to add some chemical substances into the cover materials, which act as cooling materials by controlling the solar radiation into the greenhouse. We can reduce near-infrared radiation through achieving high reflectance or low transmittance. For covering materials, common plastic films are polyethylene (PE), poly (methyl methacrylate) (PMMA), and ethylene vinyl acetate (EVA) [1,11,15], and the chemical substances are mainly divided into organic or inorganic [13,19,20]. However, organic substances have poorer dispersing ability, weather resistance, and thermal stability than that of the inorganic substances.

Passive cooling methods offer a substantial impact on energy saving due to their ability to operate without external energy [25,26,27,28,29,30,31,32,33,34,35]. Radiative cooling refers to thermal radiation from a hot object through the infrared transparency window between 8 and 13 micrometers of the atmosphere to the cold sink of outer space. Historically, radiative cooling during nighttime has been widely studied and employed for rooftop cooling. For example, Granqvist et al. [35] showed that silicon monoxide films on KRS-5 had low transmittance in 8–13 micrometers, but that cooling demand is more important during the daytime. The use of advanced photonic devices led to daytime radiative cooling well below the ambient temperature, with their high solar reflection and selective, but strong, thermal emission. In the past, Raman et al. [28] proposed a photonic radiative cooler consisting of seven alternating layers of hafnium dioxide and silicon dioxide of varying thicknesses on top of silver. It achieved cooling 5 °C below the ambient temperature. Gentle and Smith [21] used a stack of alternating layers of polymers, and a silver layer was deposited at the bottom of the polymer layers. This device achieved cooling 2 °C below the ambient temperature under direct solar irradiation exposed with no convection shield, and this was 11 °C below a quality commercial cool roof. In addition to the devices mentioned above, microstructure photonic devices, such as plasmonic structures, metallic photonic crystals, and metamaterials, were also used to improve the efficiency of radiative cooling during the daytime. However, these approaches may require stringent, precision fabrication and complex structures, which was neither cost-effective nor of a large enough production scale to apply to commerce. Polymeric photonics is a growing field attractive for economy and scalability. Yao Zhai et al. [34] manufactured a randomized glass-polymer hybrid metamaterial consisting of silicon dioxide microspheres. 

In contrast to most of the currently employed cooling methods which require energy and resources to carry heat away, radiative cooling is a passive enhancement of the Earth’s natural method of cooling itself. Thus far, radiative cooling was applied to the coating of the solar cells and roof cooling systems, but seldom employed in improving greenhouse cladding. It has great potential to combine greenhouse cladding with passive cooling to save energy and simultaneously regulate the temperature inside a greenhouse. In this study, instead of using multilayer film, we simplified the method and used silica as an addition to serve as a basis for radiation cooling. 

When transparent films made of polymer resins, such as polyethylene and polyvinyl chloride, are used in agricultural greenhouses, the water vapor condenses on the surface of the film due to the evaporation of water on the surface of the soil, crops, and plants. The water droplets refract or reflect the sunlight to form a lens, which causes light and heat to be concentrated on the local crop, causing burns to the crop. A large number of water droplets adhere to the surface of the film. This not only reduces light intensity but also increases humidity, which impacts plant growth. However, UV radiation may result in the aging of films, which affects their durability. Therefore, an anti-fog agent and light stabilizer were used in this study. The purpose of this study is to improve the plastic greenhouse cladding to regulate the temperature inside the greenhouse, mainly by passive cooling. Silica particles were added to a low-density-polyethylene matrix—which was based on formulations with low-density-polyethylene (LDPE), hindered amine light stabilizers, and anti-fogging agent—to manufacture the newly developed cladding. Silica particles were expected to improve thermal radiation performance to assist newly developed cladding in reducing the temperatures inside a greenhouse. 

## 2. Materials and Methods 

### 2.1. Materials and Preparing Plastic Films

The materials used in the experiment are low-density polyethylene (LDPE, USI Corporation, Kaohsiung, Taiwan), silica (SiO_2_, Oriental Silicas Corporation, Taipei, Taiwan), hindered amine light stabilizers (HALS, UA944, Elite Chem Co., Ltd., Taipei, Taiwan), and anti-fogging agent (GY-NA-3000, Go Yen Chemical Industrial Co., Ltd., Kaohsiung, Taiwan).

HALS (30 g) and GY-NA-3000 (36 g) were separately mixed with LDPE, and the two mixtures were heated to 200 °C and underwent extrusion molding to produce two kinds of monolayer films: LDPE + H and LDPE + G. 

Silica (30 g) was treated with HALS (30 g) used as a UV stabilizer, then mixed with LDPE (3000 g) and GY-NA-3000 (36 g) and heated to 200 °C. Finally, it underwent extrusion molding to produce monolayer plastic films with a thickness of 0.16–0.18 mm. This was one kind of newly developed cladding (1%S monolayer). The other newly developed cladding (1%S double) was prepared as follows. Silica (30 g) was first treated with HALS (30 g). Next, the treated silica was mixed with LDPE (3000 g). At the same time, LDPE was mixed with GY-NA-3000 (36 g) and UA944 (30 g) to produce the other kinds of mixtures. Then, two kinds of mixtures were separately heated to 200 °C to get molten mixtures. Finally, they met at the extrusion die and they underwent extrusion molding to produce double layer plastic films with the thickness of 0.16–0.18 mm 

### 2.2. Characterization of Prepared Plastic Films

An atomic force microscope (AFM, JPK, Axiovert200, Berlin, Germany) was used to determine the roughness of the surface of plastic films in intermittent contact mode. The AFM probes were Tap 150Al-G silicon probes, which were purchased from Budget Sensors. The transmittance spectra of the films were measured by a fiber optic spectrometer (Ocean Optics, USB4000-VIS-NIR, Winter Park, CO, USA). Ten points on the cladding were taken to get a range of the transmittance of the newly developed claddings. The transmittance and reflection were found by integrating the areas of the spectrum using 

(1) $$\;VIS\;\mathrm{transmittance}\;\mathrm{or}\;\mathrm{reflection}\;=\;\;\frac{\int_{400}^{780}\;\;Is\;\left(\lambda \right)\;d\lambda \;}{\int_{400}^{780}Il\left(\lambda \right)d\lambda \;}$$

(2) $$\mathrm{NIR}\;\mathrm{transmittance}\;\mathrm{or}\;\mathrm{reflection}\;=\;\frac{\int_{780}^{1035}\;\;Is\;\left(\lambda \right)\;d\lambda \;}{\int_{780}^{1035}Il\left(\lambda \right)d\lambda \;}\;\;\;$$

In Equations (1) and (2), Is represents the intensity of newly developed cladding in the spectrum and Il is the intensity of light in the spectrum.

We characterized the spectroscopic performance of newly developed cladding in infrared (4 to 20 µm) by using Fourier-transform infrared spectroscopy (FTIR, SHIMADZU, 8400, Kyoto, Japan). An inverted metallurgical microscope (OLYMPUS, GX51, Tokyo, Japan) was used to observe the dispersion of silica in the films, and a scanning electron microscope (SEM, JEOL Ltd., JSM-6390LV, Tokyo, Japan) was used to inspect the cross-section of the films. The tensile strength and the maximum elongation of films were measured using a tensile strength tester (GOTECH testing machines INC., AI-2500, Taichung, Taiwan) according to ASTM D638 (type IV). The films were also used as the cladding to construct a simulated greenhouse. The temperatures of the simulated greenhouse were recorded in real time with thermocouples (TECPEL Co., LTD., DTM-319A, New Taipei, Taiwan). The observed temperatures were used for evaluating the cooling performance of the newly developed cladding. 

## 3. Results and Discussion

### 3.1. AFM Analysis of the Samples

**Figure 1:**
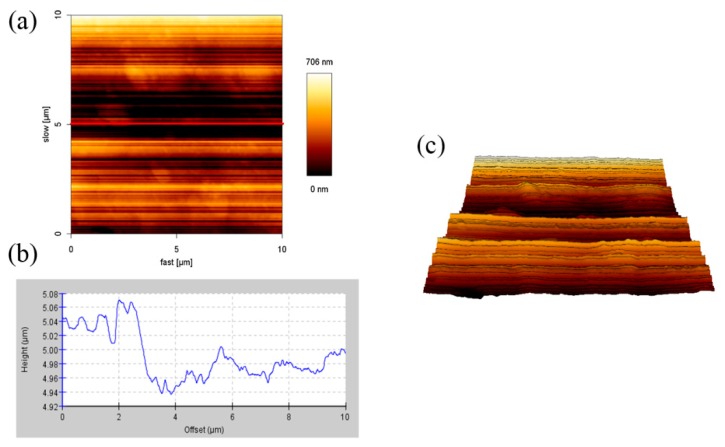
Atomic force microscope (AFM) images (10 × 10 µm) of 1% SiO_2_ monolayer films: (**a**) A height image; (**b**) cross-section height profiles along the line in (**a**), and (**c**) a 3D reconstruction image.

When light irradiates to the cladding, penetration, reflection, and absorption will occur. If the surface of the cladding is not smooth enough, it will affect the penetration of the cladding. AFM was used to measure the roughness of the surface and observe the height the cladding. Figure 1 shows the AFM surface height images, cross-section profiles, and the 3D reconstruction image of 1% SiO_2_ monolayer films (1%S monolayer). The cross-section height profiles are along the black line in the AFM height image. The height of the 1%S monolayer was 0.02–0.11 μm, and the width of the ups and downs in the cross-section height profiles were 1 to 2 μm and considered as silica particles. However, the surface roughness of this area was 0.16 μm. 

**Figure 2:**
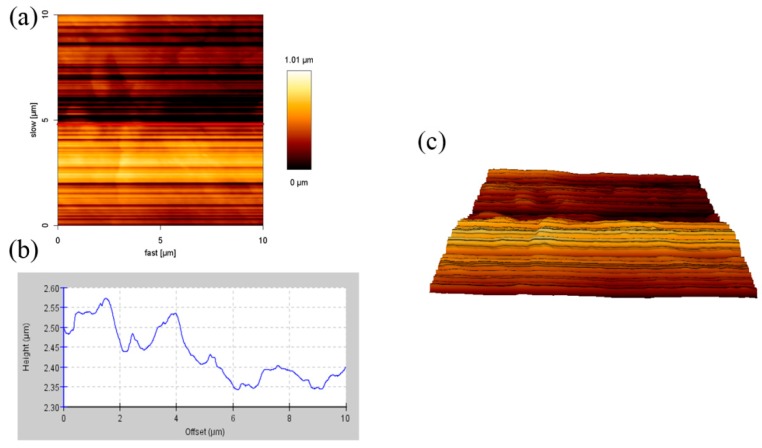
AFM images (10 × 10 µm) the side of anti-fogging agent of 1% SiO_2_ double layer films: (**a**) A height image; (**b**) cross-section height profiles along the line in (**a**), and (**c**) a 3D reconstruction image.

**Figure 3:**
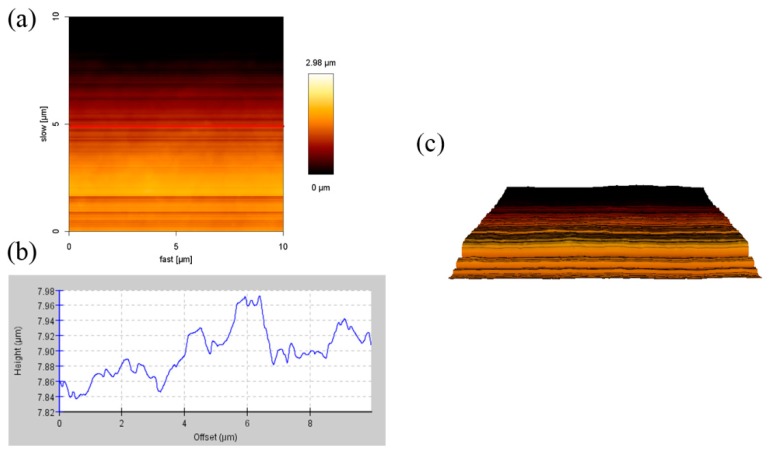
AFM images (10 × 10 µm) of the side of the treated silica of 1% SiO_2_ double layer films: (**a**) A height image; (**b**) cross-section height profiles along the line in (**a**), and (**c**) a 3D reconstruction image.

Figure 2 and Figure 3 showed the AFM images of 1% SiO_2_ double layer films (1%S double) with different sides. Their heights were 0.02–0.12 μm and 0.02–0.09 μm, respectively, and the surface roughness of the area was 0.23 and 0.68 μm, respectively. Compared to the side of the treated silica of the 1%S double films, the surface roughness on the side of the anti-fogging agent (0.23 µm) was small. This is because the melting point of GY-NA-3000 and UA944 is 70 °C and 100–135 °C, respectively. During the manufacturing process of the films, they were heated to 200 °C. Therefore, there were theoretically no particles on the side of the anti-fogging agent. According to the results from Figure 5, after adding silica, the transmittance and reflection of the films did not decrease. Therefore, the effect of surface roughness on penetration was ignored in this experiment.

### 3.2. Mechanical Properties of the Samples

The tensile strength and the maximum elongation of the samples (size: 25 mm) were measured and the results are shown in Table 1. As shown, LDPE has low strength—about half of the commercial agricultural PE films. In comparison, commercial agricultural PE film had the greatest tensile strength and maximum elongation due to having fewer branches. When silica is added (1%S monolayer and 1%S double), the tensile strength and maximum elongation were increased as much as 60–70%. Their performances were still poorer than PE, but these additions did not make the mechanical properties worse.

### 3.3. UV-VIS Spectra of the Samples

**Figure 4:**
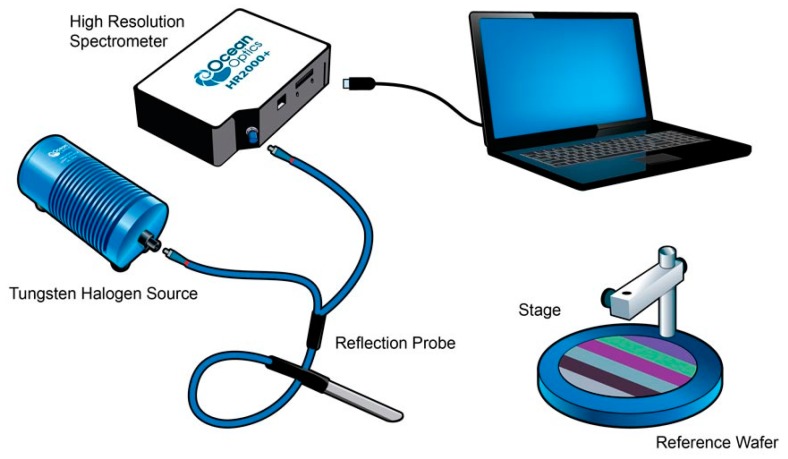
An optical device for measuring ultraviolet-visible radiation (UV-VIS) spectra.

**Figure 5:**
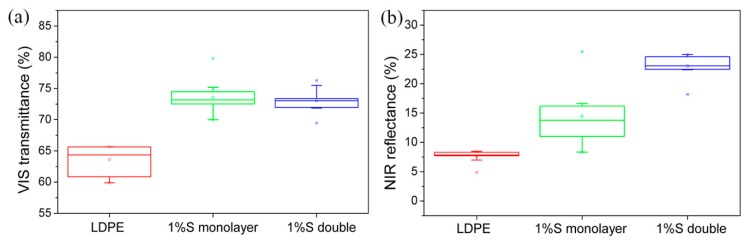
(**a**) A range of the visible radiation (VIS) transmittance of low-density-polyethylene (LDPE), 1%S monolayer, and 1%S double; (**b**) A range of the near-infrared radiation (NIR) reflectance of LDPE, 1%S monolayer, and 1%S double.

We took ten points on the films and used Ocean Optics to measure the spectral reflectance in the VIS-NIR region. Figure 4 is the optical device for measuring UV-VIS spectra. Then, we used origin Pro8 to integrate the areas of the spectra to get the transmittance and reflectance, where the difference between the measurement of transmittance and reflection is that the optical fibers and installation positions are different. According to Equations (1) and (2), a range of the VIS transmittance and the NIR reflectance of the different films by measuring ten different points on the films was shown in Figure 5. LDPE had 63% VIS transmittance. After adding UA994, GY-NA-3000, and silica, the VIS transmittance increased to about 73% (Figure 5a). Next, the NIR reflectance of LDPE was only 7% and that of the 1%S monolayer was 14%. The VIS transmittance and the NIR reflectance of 1%S monolayer and 1%S double were better than that of LDPE. High NIR reflectance is due to the NIR reflection by silica. However, although the same amount of silica was added, 1%S had a doubly increased NIR reflectance of approximately 23%. The dispersion of the particles may influence the reflectance, and double layer films dispersed better than monolayer ones, as proven in Figure 8. Therefore, 1%S double had the biggest NIR reflectance. The commonalities between VIS transmittance and the NIR reflectance was that the range of 1%S double was smaller than that of 1%S monolayer because of the good dispersion in double layer films. 

### 3.4. FT-IR Spectrum of the Samples

**Figure 6:**
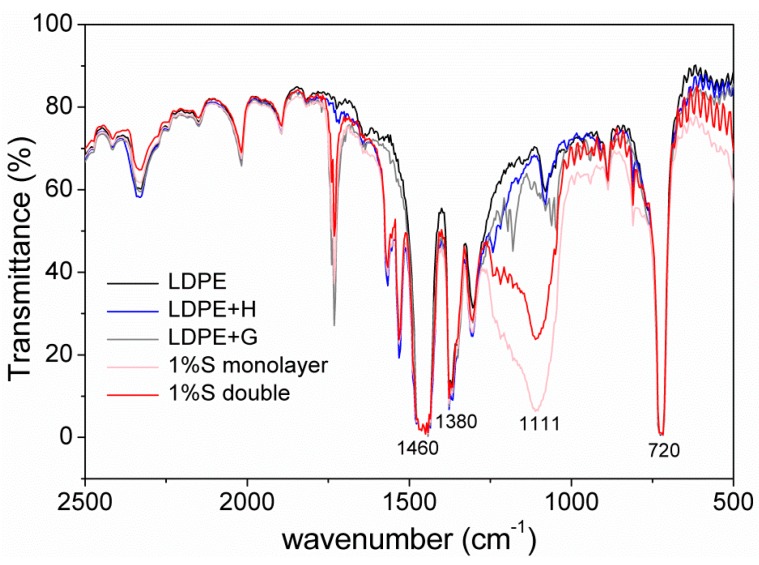
Fourier-transform infrared spectroscopy (FT-IR) spectra of different films.

Figure 6 showed an FT-IR spectrum between 500 and 2500 cm^−1^ (4 to 20 µm) of different films. It was clear that there was no change when LDPE had added HALS (LDPE + H) and GY-NA-3000 (LDPE + G). A strong absorption peak near 1000 cm^−1^ appeared after adding silica particles. The characteristic peaks at 720, 1380, and 1460 cm^−1^ belong to –CH_2_– in polyethylene. The peaks near 1111 cm^−1^ were attributed to SiO_2_.

**Figure 7:**
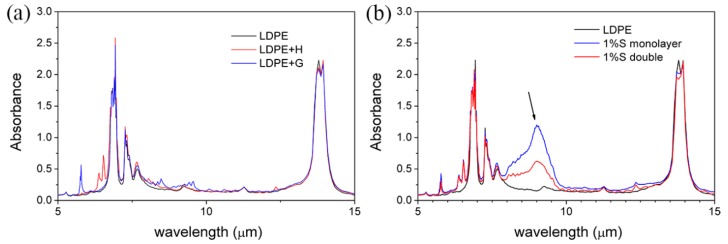
(**a**) An absorption spectrum of LDPE, LDPE + H, and LDPE + G; (**b**) An absorption spectrum of LDPE, 1%S monolayer, and 1%S double.

If the electromagnetic waves radiated by the object cannot be absorbed by the atmosphere, then it can penetrate the atmosphere and dissipate energy in outer space. It is well known that the Earth’s atmosphere has a highly transparent window in the infrared (IR) wavelength range between 8 and 13 μm. Thus, whether the newly developed cladding has an absorption peak within 8–13 μm is very important. In order to observe this precisely, Figure 6 was converted into an absorption spectrum and *x*-axis units were converted to micrometers, as shown in Figure 7. Firstly, LDPE + H and LDPE + G had no clear change in the overall curve in Figure 7a, and this result represented that UA994 and GY-NA-3000 were useless for passive cooling. In Figure 7b, a powerful absorption peak near 9 µm in the 1%S monolayer and 1%S double appeared because of the characteristics of silica. The absorption of the 1%S monolayer is evidently stronger than that of 1%S double. During the manufacturing processes of the films, all additions were mixed and extruded into a 1%S monolayer with a thickness of 0.16–0.18 mm. However, there were two layers in 1%S double, with only half the silica content (0.08–0.09 mm) at the same thickness. This reason caused the 1%S monolayer to have a bigger absorption at 9 µm than 1%S double.

### 3.5. Inverted Metallurgical Microscope Images of the Samples

**Figure 8:**
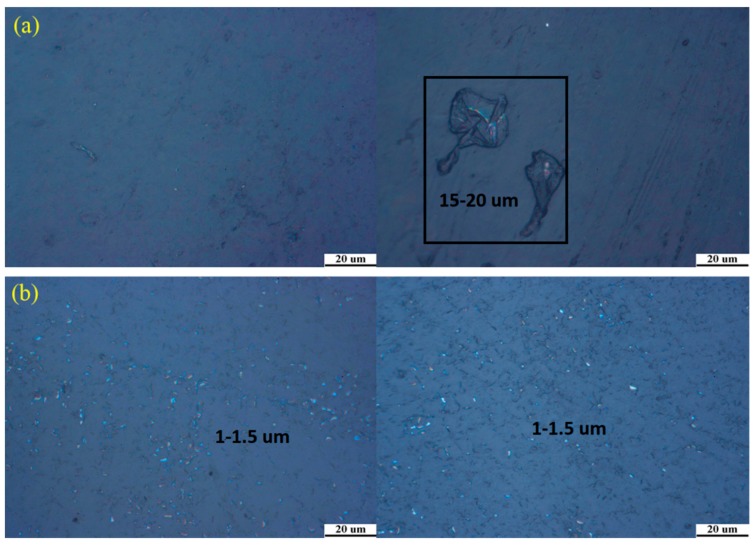
Inverted metallurgical microscope images of (**a**) 1%S monolayer and (**b**) 1%S double.

Dispersion will affect the properties of the materials, so it is very important to study the dispersion of silica particles in the films. Figure 8 showed inverted metallurgical microscope images of the 1%S monolayer and 1%S double in different areas. Silica particles are a white powder and their size is around 1300 nm. Compared to 1%S double, the 1%S monolayer white points, as shown in Figure 8a, did not evenly distribute. In the black box, the size of the white area was about 15–20 µm. This result indicated that silica particles were reunited in the films. However, in Figure 8b, it can clearly be seen that the size of the white points were within the range of 1–1.5 µm, which suggests that silica particles dispersed well in 1%S double.

### 3.6. SEM Images of the Samples

**Figure 9:**
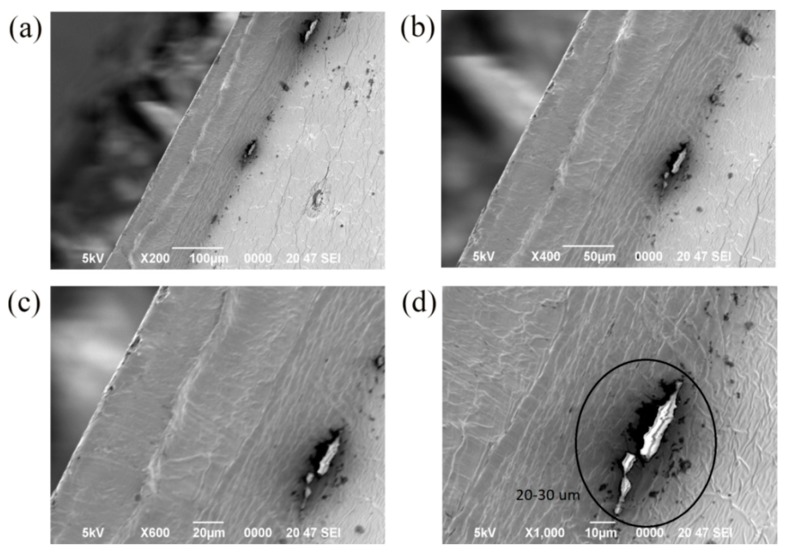
SEM images of 1%S monolayer with different magnifications: (**a**) 200×; (**b**) 400×; (**c**) 600×; and (**d**) 1000×.

**Figure 10:**
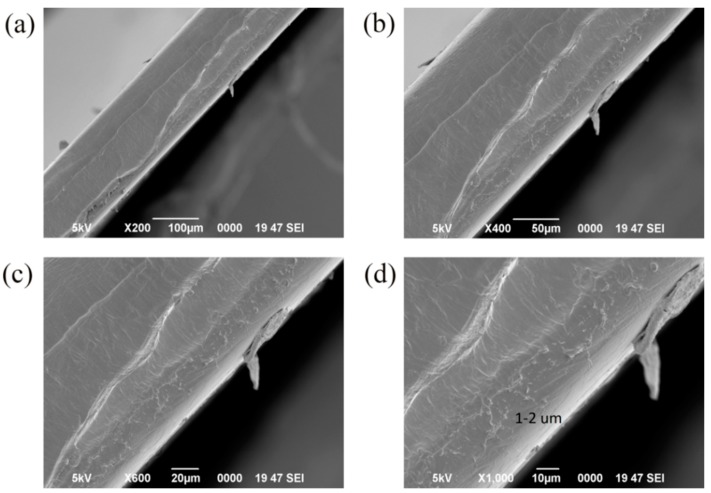
SEM images of 1%S double with different magnifications: (**a**) 200×; (**b**) 400×; (**c**) 600×; and (**d**) 1000×.

To understand the dispersion of silica particles in the films, the cross-sections of the films were observed with a SEM. Figure 9 and Figure 10 were the SEM images of the cross-section of the 1%S monolayer and 1%S double, and a–d were at the same position in the 1%S monolayer or 1%S double with different magnifications. There were many small white points in Figure 10d and their sizes were approximately 1 to 2 µm. This is consistent with the size of silica particles. This demonstrates that silica particles distribute uniformly with less aggregation in 1%S double. However, in Figure 9d, the size of the white area in the black circle was 20–30 µm, and it was obvious that the aggregation of silica particles presented in the 1%S monolayer. Both of the results from the inverted metallurgical microscope and SEM showed that silica particles in the 1%S monolayer dispersed more poorly than that of 1%S double. Therefore, we used 1%S double for the cooling performance experiment. 

### 3.7. Cooling Performance

**Figure 11:**
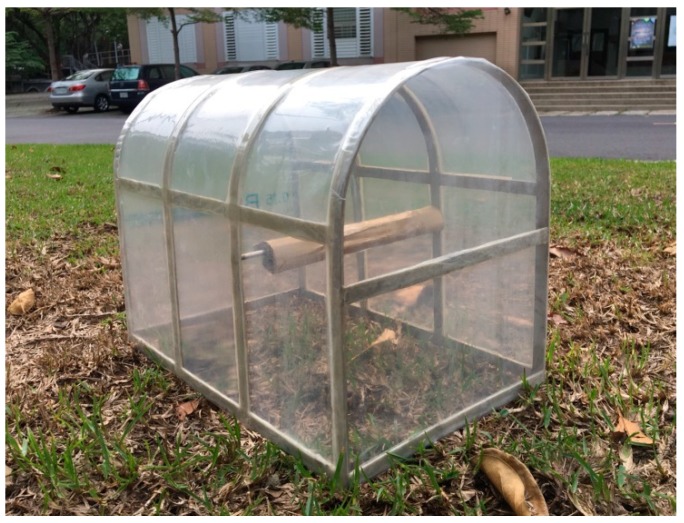
A simple simulated greenhouse.

**Figure 12:**
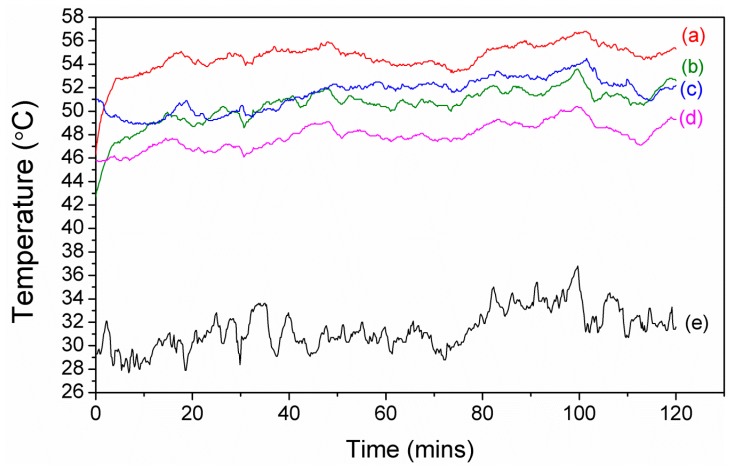
(**a**) Air temperature trend of polyethylene (PE) film inside the greenhouse; (**b**) Temperature trend of PE film on the cladding surface; (**c**) Air temperature trend of 1%S double inside the greenhouse; (**d**) Temperature trend of 1%S double on the cladding surface; (**e**) Ambient air temperature.

In order to determine the cooling performance of 1%S double, we set up a simple simulated greenhouse (32 × 20 × 25 cm, Figure 11) and measured the temperature inside the greenhouse, on the cladding, and the ambient temperature from 11 a.m. to 1 p.m. in Chiayi, Taiwan. PE was used as a comparison. Between curve (a) and curve (c) in Figure 12, under about 35 °C ambient conditions, the air temperature inside the greenhouse had a maximum temperature difference of 5.4 °C and an average difference of 3.1 °C. However, between curve (b) and curve (d) in Figure 12, the temperature on the cladding had a maximum temperature difference of 3.6 °C and an average difference of 2.6 °C. These results demonstrated that 1%S double possessed good cooling performance, and this was contributed to not only by the high NIR reflection, but also good passive cooling performance, which resulted from emitting radiation between 8–13 µm through the atmosphere window by silica, as shown in Figure 7. It can be explained that the addition of the treated silica was effective in lowering the heat loading of the greenhouse through greater NIR reflection and radiative cooling.

## 4. Conclusions

In this study, new greenhouse cladding (1% SiO_2_ monolayer films and 1% SiO_2_ double layer films) made of a LDPE-matrix composite was developed. The newly developed cladding has the following advantages.

1. The VIS transmittance and the NIR reflectance of 1% SiO_2_ monolayer films and 1% SiO_2_ double layer films were better than that of LDPE.
2. The images of inverted metallurgical microscope and SEM show that the silica particles in 1% SiO_2_ double layer films distributed more uniformly than that of monolayer films. It is suggested that the double layer film is the better choice.
3. FT-IR spectra depict that strong absorption occurs in 9 µm for both 1% SiO_2_ monolayer films and 1% SiO_2_ double layer films.
4. The mechanical properties of the raw LDPE used in this study are half those of the commercial agricultural PE films. However, with the addition of silica, the mechanical properties of the newly developed films increased as much as 60–70%.
5. Under 35 °C ambient conditions, the inner temperature of the simulated greenhouse with the 1% SiO_2_ double layer films was 3 to 5 °C less than that of the simulated greenhouse with the commercial agricultural PE cladding. The temperature on the 1% SiO_2_ double layer films was reduced 2 to 4 °C in comparison with that of the commercial PE cladding. This is evidence that radiative cooling is helpful in the cooling performance of the greenhouse.

## Figures and Tables

**Table 1:** Mechanical properties of the samples.

Samples	Tensile Strength (MPa)	Maximum Elongation (%)
PE	21.32	1506.64
LDPE	10.39	997.43
1%S monolayer	16.89	1244.02
1%S double	16.62	1259.80
